# Neuronal Morphological Model-Driven Image Registration for Serial Electron Microscopy Sections

**DOI:** 10.3389/fnhum.2022.846599

**Published:** 2022-05-05

**Authors:** Fangxu Zhou, Bohao Chen, Xi Chen, Hua Han

**Affiliations:** ^1^School of Future Technology, University of Chinese Academy of Sciences, Beijing, China; ^2^Institute of Automation, Chinese Academy of Sciences, Beijing, China; ^3^School of Artificial Intelligence, University of Chinese Academy of Sciences, Beijing, China; ^4^Center for Excellence in Brain Science and Intelligence Technology, Chinese Academy of Sciences, Shanghai, China; ^5^National Laboratory of Pattern Recognition, Institute of Automation, Chinese Academy of Sciences, Beijing, China

**Keywords:** neuronal structure, spherical deformation model, image registration, registration accuracy, serial section electron microscopy

## Abstract

Registration of a series of the two-dimensional electron microscope (EM) images of the brain tissue into volumetric form is an important technique that can be used for neuronal circuit reconstruction. However, complex appearance changes of neuronal morphology in adjacent sections bring difficulty in finding correct correspondences, making serial section neural image registration challenging. To solve this problem, we consider whether there are such stable "markers" in the neural images to alleviate registration difficulty. In this paper, we employ the spherical deformation model to simulate the local neuron structure and analyze the relationship between registration accuracy and neuronal structure shapes in two adjacent sections. The relevant analysis proves that regular circular structures in the section images are instrumental in seeking robust corresponding relationships. Then, we design a new serial section image registration framework driven by this neuronal morphological model, fully utilizing the characteristics of the anatomical structure of nerve tissue and obtaining more reasonable corresponding relationships. Specifically, we leverage a deep membrane segmentation network and neural morphological physical selection model to select the stable rounded regions in neural images. Then, we combine feature extraction and global optimization of correspondence position to obtain the deformation field of multiple images. Experiments on real and synthetic serial EM section neural image datasets have demonstrated that our proposed method could achieve more reasonable and reliable registration results, outperforming the state-of-the-art approaches in qualitative and quantitative analysis.

## 1. Introduction

Connectomics is an important research field in exploring and understanding the nanoscale neuronal structures of the brain, which is of great significance for research on neuronal function, neurogenic diseases, and brain-inspired intelligence (Paul Alivisatos et al., 2012; Luo, 2017; Sun and Du, 2018). Serial sections electron microscopy (ssEM) imaging is one of the important techniques to obtain three-dimensional (3*D*) neural tissue image data at nanometer resolution (Wanner et al., 2015). For serial sections imaging (Kasthuri et al., 2007), the sections of brain tissues are cut into 30–50 nm-thick by an ultramicrotome and collected onto conductive supporters, such as electron opaque support tape (e.g., ATUM-SEM Hayworth et al., 2014) or metal support grids (e.g., ssTEM Harris et al., 2006). The sections can be imaged in parallel to save data acquisition time, which facilitates studies of large-scale neural circuit reconstruction. However, such brain imaging technique inevitably brings the loss of 3*D* integrity. Therefore, the image registration technique is crucial for getting these serial two-dimensional (2*D*) EM images into 3*D* volumetric form for subsequent neural circuit reconstruction and analysis (Briggman and Bock, 2012).

Images registration of neural tissue sections is not an easy task. This is because the neuronal structures change dynamically along the sections. In addition, sections cut sequentially at specific intervals are unique and contiguous. This means that the contents of the adjacent images are not identical but similar, and the similarity depends on neuronal structure variation and section thickness. Most existing image registration approaches consist of three steps: feature extraction, feature matching, and regularization using a specified transformation model (e. g. rigid, affine, diffeomorphic models, etc.). Such complex appearance changing of neural images brings enormous challenges for this pipeline's first two steps (feature extraction and matching). The key to solving serial section neural image registration is finding robust and reliable correspondences under such changeable appearance circumstances.

There are several conventional nonlinear registration methods using hand-crafted features for aligning serial section images, such as scale-invariant feature transform (SIFT) (Lowe, 2004) and block-matching. Some open-source ssEM image registration tools use these types of hand-crafted features, such as elastic alignment (Saalfeld et al., 2012) (available *via* TrakEM2 Albert et al., 2012). However, these types of methods could not guarantee that correct correspondence is detected in the adjacent sections with considerable appearance changing. Most of them (Wang et al., 2015) choose one of the serial images as the reference image and then sequentially perform forward or backward pairwise image registration. As a result, these methods may introduce error accumulation and propagation, which leads to artificial deformation for images that are far away from the reference image. Recently, many state-of-the-art results in deep learning have been proposed for computer vision problems. Some researchers have drawn from natural image registration methods and apply to ssEM image registration. They used the data-driven deep learning method to measure the similarity between sections and minimize the square error between the target image and the registered source image to obtain the deformation field. Yoo et al. (2017) proposed an end-to-end trained 2D convolutional autoencoder (Hinton and Salakhutdinov, 2006) to generate feature maps and then combined them with a spatial transformer network (STN) (Jaderberg et al., 2015) to obtain the vector field. Mitchell et al. (2019) leveraged a siamese convolutional network to encode images and extract features, and a coarse-to-fine recursion trained alignment module to transform the source image. Despite the above significant progress, serial section neural image registering methods do not take full advantage of neuronal morphological characteristics. Due to the lack of utilization of characteristics of image content and the variation adaptability, they can hardly capture the reliable corresponding relationship in adjacent images of neural tissues well, which may lead to registering failures. Note that there exists a strong spatial morphological relationship between the appearances of neural structures in consecutive sections. Therefore, we propose that serial image registering can benefit from the neuronal morphological modeling of neuronal structures in neural images.

In addition, instead of using image information, some methods employ the straightforward way to build the corresponding relation, in which implants are utilized as registration landmarks in the biological tissues (Pauchard et al., 2004; Yavariabdi et al., 2013). Such landmarks are steady, regular, and unvarying structures in the adjacent images. They are directly selected as correspondences to register biological tissues, reducing the difficulties of seeking robust and reliable correspondence in significant appearance-changing neural images. However, the method of using implants as correspondences for registration is limited by additional experimental operations. Therefore, we consider whether there are such relatively stable structures in neural images.

Motivated by the above observations, in this work, we model the nerve morphological structure to investigate the stable structure as the "landmarks" for serial neural image registration. In previous work (Bohao et al., 2022), the authors used the spherical deformation model to simulate neurons to investigate the effects of section thickness and neuronal structure size on the ssEM registration accuracy. Inspired by this work, we use the spherical deformation model to simulate the local neuronal structure, generate synthetic pre-aligned image data, and mathematically analyze the relationship between registration accuracy and neuronal structure shapes in two adjacent sections. These findings presented that registration accuracy is positively correlated with neuronal structure roundness, or in other words, the more regular the shape of neuronal structure, the more accurate it is to register adjacent sections. Based on the conclusion of neuronal morphological modeling analysis, we go a step further by proposing a novel framework for serial neural image registration, consisting of a neuronal morphological model-driven region selection module, correspondence extraction and global optimization module, and image deformation module. Specifically, a mask is obtained in the neuronal morphological model-driven region selection module as the adaption guidance by a deep segmentation network and neural morphological physical selection model. With this mask, the feature can be adaptively extracted by focusing on the stable region and paying less attention to changing region (unstable structure). In correspondence extraction and global optimization module, the reliable correspondences between the adjacent sections are extracted by the local feature description method SIFT-flow (Liu et al., 2011). Then, the corresponding points are adjusted globally (the whole sequence) based on an energy function to satisfy the position consistency of the extracted correspondences, reducing error propagation in computing multiple images, and preserving section continuities. Finally, each section image is deformed according to the position adjustment result of the correspondences in the image deformation module. We validate the effectiveness and efficiency of our approach on two datasets of serial EM sections.

To sum up, the main contributions of this work are as follows:

**(1)** We model the neuronal morphology by spherical deformation model. The relationship between registration accuracy and neuronal structure shapes in two adjacent sections is mathematically analyzed. The relevant analysis proves that more regular circular structures in the section images should be selected as "landmarks."

**(2)** We design a new serial section image registration framework driven by neuronal morphological model, fully utilizing the characteristics of the anatomical structure of nerve tissue and obtaining more reasonable corresponding relationship. Our proposed framework consists of a neuronal morphological model-driven region selection module, correspondence extraction and global optimization module, and image deformation module.

**(3)** Experimental results on different datasets show that the proposed register performs significantly better than the state-of-the-art algorithms, achieving more genuine deformation results.

The remainder of this paper is organized as follows. Section 2 briefly describes the datasets used in this paper, mathematically analyses neural image registration accuracy by neuronal morphology model, and presents the proposed registration framework. Section 3 provides experiment results on a real and synthetic dataset. Finally, we conclude the paper in Section 4 with a discussion of our findings and future research directions.

## 2. Materials and Methods

### 2.1. Data Preparation

In this subsection, we briefly describe datasets used in this paper. We use three biological datasets and one synthetic dataset to validate our serial section image registration framework and verify the registration accuracy theory. We first introduce the biological datasets, then describe how to model a tubular neuron as local sphere-like structures, and generate synthetic pre-aligned image data.

#### 2.1.1. Serial Section Neural Images Acquisition

**Drosophila ssTEM (DST) dataset:** These data were released by Gerhard et al. (2013), containing 20 sections of a Drosophila brain from serial section transmission electron microscopy (ssTEM). The section thickness is 40-50 *nm*. The resolution is 1, 024 × 1, 024 pixels with a 4.6 *nm* pixel size in the *xy* plane.

**Drosophila FIB-SEM (DFS) dataset:** These data include 31 serial sections of a Drosophila brain acquired using FIB-SEM (Knott et al., 2008). The solution is 6, 684 × 6, 516 pixels with a 9.15 *nm* pixel size in the *xy* plane. Because the serial EM sections are highly anisotropic in resolution, the section thickness is set to 100 *nm*. The sections of this dataset are imaged *in situ*, while due to the imaging parameter drift, a little shift has existed. It could be corrected by simple linear alignment. The finely adjusted FIB-SEM images are often regarded as the ground truth in serial section image registration and can introduce synthetic deformation to simulate the deformation received by real slices. We can quantitatively evaluate registration methods by comparing the effect of aligning these artificially deformed images.

To generate the artificially deformed images, each section is first rotated by a random angle and shifted in a random displacement. The random angle is uniformly distributed between –90 and 90 degrees, and the random displacement is uniformly distributed between –100 and 100 pixels. Then, in order to simulate the nonlinear distortion during the section cutting, we further distort every five sections artificially using MLS (Schaefer et al., 2006) by displacing four control points. The image is evenly divided into four parts by cross line, each control point is randomly selected at random positions within the range of more than 50 pixels from the edge of each part.

**Drosophila ssTEM dataset from CREMI challenge (DST-CREMI):** CREMI dataset is from an adult Drosophila melanogaster brain and imaged with ssTEM. It consists of 125 serial EM images with the voxel resolution size of 4 × 4 × 40*nm* and pixel resolution of 1, 250 × 1, 250. CREMI datasets have undergone strict manual registration and neurite membrane annotation, which can be treated as the ground truth of the EM image to be registered. We follow the process of Yoo et al. (2017) to generate synthetic deformation data: deformed using the TPS method (Bookstein, 1989) by several random vectors on random positions. The random vectors were sampled from the normal distribution with a zero mean value, and the random positions were uniformly distributed in space.

#### 2.1.2. Synthetic Dataset

**Figure 1:**
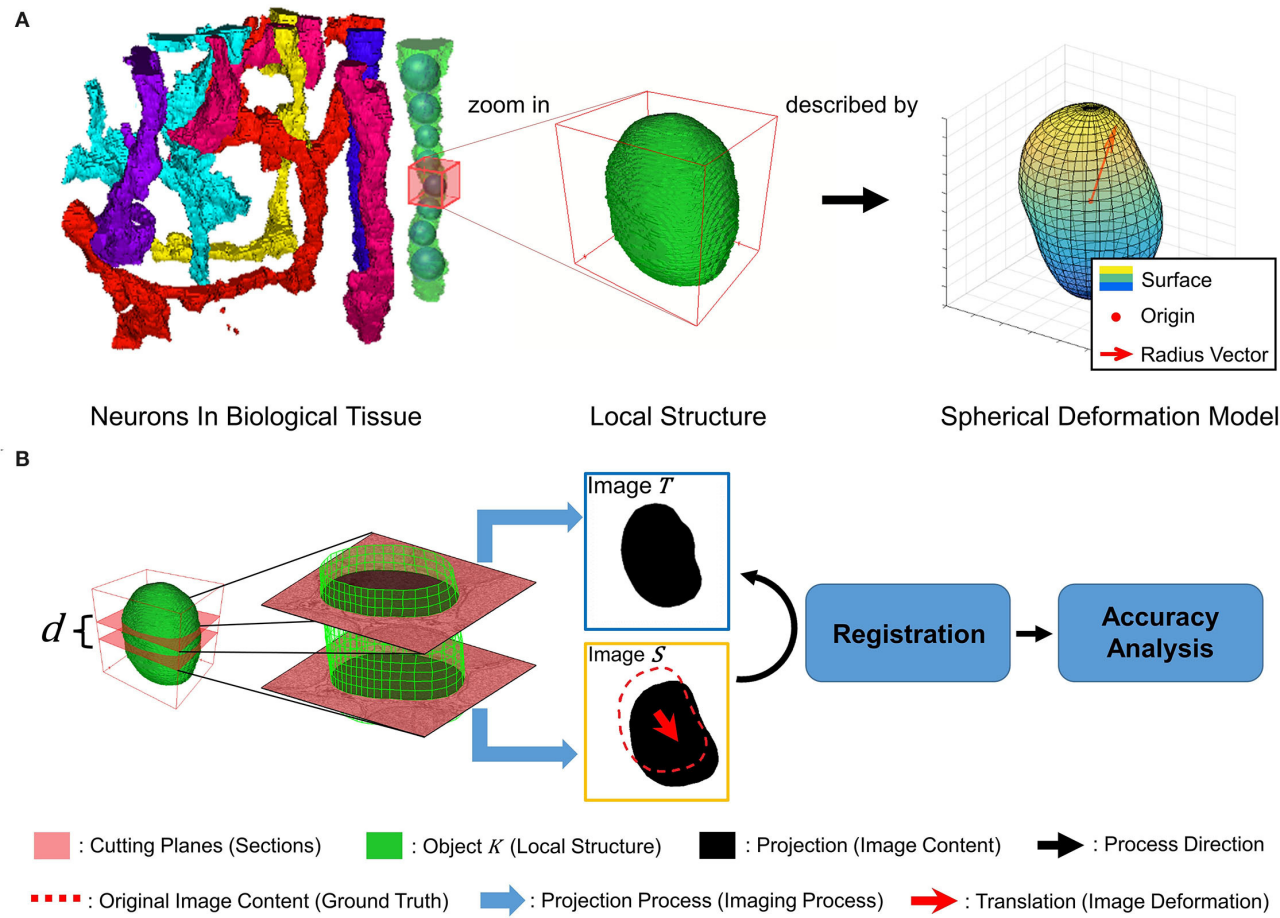
**(A)** A tubular-like neuron is modeled as a sequence of local sphere-like structures described by a spherical deformation model with different parameters. **(B)** Illustration of simulated imaging, image deformation, registration, and accuracy analysis process.

Inspired by previous work modeled vessels as the envelope of a family of spheres in Wang et al. (2020), we model the tubular neuronal structure in biological tissue as a series of local sphere-like structures, as shown in Figure 1A. Each local structure is described by the spherical deformation model, which is analyzed in detail in Hobolth (2003). The spherical deformation model has been successfully used to fit the surfaces of neurons in the human hippocampus. Further, we intend to simulate the registration of local structure *K* in adjacent sections in neuronal circuit reconstruction. The local structure *K* is cut into two parallel planes with distance *d*, regarded as adjacent sections. *d* is regarded as section thickness. The projections of *K* on the cutting planes are regarded as image content. After the projecting process, i.e., the simulated imaging process, we add a translation to one image, regarded as image deformation during the slicing and imaging process. Then, we register these two images and analyze the relationship between registration accuracy and neuronal structure shape. Figure 1B illustrates the above process.

We generated a synthetic dataset based on the spherical deformation model with the above description of modeling local structure and simulated registration process. Now, we briefly introduce the spherical deformation model for the following accuracy analysis and synthetic data generation. For a local structure *K*, the spherical deformation model selects a point *z* ∈ *K* as origin and describes the surface of *K* by spherical coordinates as {*z* + *r*(θ, ϕ)ω(θ, ϕ) : 0 ≤ θ < 2π, 0 ≤ ϕ ≤ π}. The unit direction vector ω(θ, ϕ) = (cosθ sinϕ, sinθ sinϕ, cosϕ) is the vector on the unit sphere with polar longitude θ and polar latitude ϕ, and *r*(θ, ϕ) is the distance from *z* to the surface of *K*. For simplicity, we consider the normalized radius $r\left(\theta ,\phi \right)/\bar{r}$, where $\bar{r}$ is the mean radius length. The normalized radius function is written as follows


(1) $$\begin{array}{l} r\left(\theta ,\phi \right)=1+\overset{\infty }{\underset{n=2}{\sum }}\overset{n}{\underset{m=-n}{\sum }}a_{n}^{m}\varphi_{n}^{m}\left(\theta ,\phi \right), \end{array}$$

where $\varphi_{n}^{m}\left(\theta ,\phi \right)$ is the spherical harmonic function. The spherical harmonics are given by the following equations


$$\begin{array}{l} \varphi_{n}^{m}(\theta ,\phi )=\{\begin{array}{ll} k_{n}^{\left|m\right|}P_{n}^{\left|m\right|}(\cos\phi )\cos m\theta , & m=-n,\ldots ,-1\\ k_{n}^{0}P_{n}^{0}(\cos\phi ), & m=0\\ k_{n}^{m}P_{n}^{m}(\cos\phi )\sin m\theta , & m=1,\ldots ,n, \end{array} \end{array}$$

where $k_{n}^{m}$ is normalizing constant and $P_{n}^{m}$ is the associated Legendre function of the first kind. In the normalized radius function, each coefficient $a_{n}^{m}$ of spherical harmonic function is modeled as Gaussian random variables subject to $N\left(0,\lambda_{n}^{m}\right)$. The deformation model supposes there is stationarity on *K*, which is obtained by assuming $\lambda_{n}^{m}=\lambda_{n},\;n\;\geq \;2,\;m\;=\;-n,\ldots ,n.$ Besides, the covariance of vectors *r*(θ_1_, ϕ_1_) and *r*(θ_2_, ϕ_2_) on *K* is as follows:


(2) $$\begin{array}{l} \begin{array}{c} Cov\left(r\left(\theta_{1},\phi_{1}\right),r\left(\theta_{2},\phi_{2}\right)\right)=\sum_{n=2}^{\infty }\lambda_{n}\sum_{m=-n}^{n}\varphi_{n}^{m}\left(\theta_{1},\phi_{1}\right)\varphi_{n}^{m}\left(\theta_{2},\phi_{2}\right)\\ =\sum_{n=2}^{\infty }\lambda_{n}{\left(k_{n}^{0}\right)}^{2}P_{n}\left(\cos\psi \right), \end{array} \end{array}$$

where cosψ = ω(θ_1_, ϕ_1_)·ω(θ_2_, ϕ_2_), and ψ represents the spatial angle between *r*(θ_1_, ϕ_1_) and *r*(θ_2_, ϕ_2_). The spherical deformation model also supposes λ_*n*_ decrease according to


(3) $$\begin{array}{l} 1/\lambda_{n}=\alpha +\beta (n^{p}-2^{p}),\\ n\;\geq \;2,p\;>\;2,\alpha ,\beta \;>\;0. \end{array}$$

The pre-defined parameter *p* determines the smoothness of *K*, while the other two pre-defined parameters α and β determine the global and local shape, respectively. More information about the spherical deformation model can be found in Hobolth (2003).

**Figure 2:**
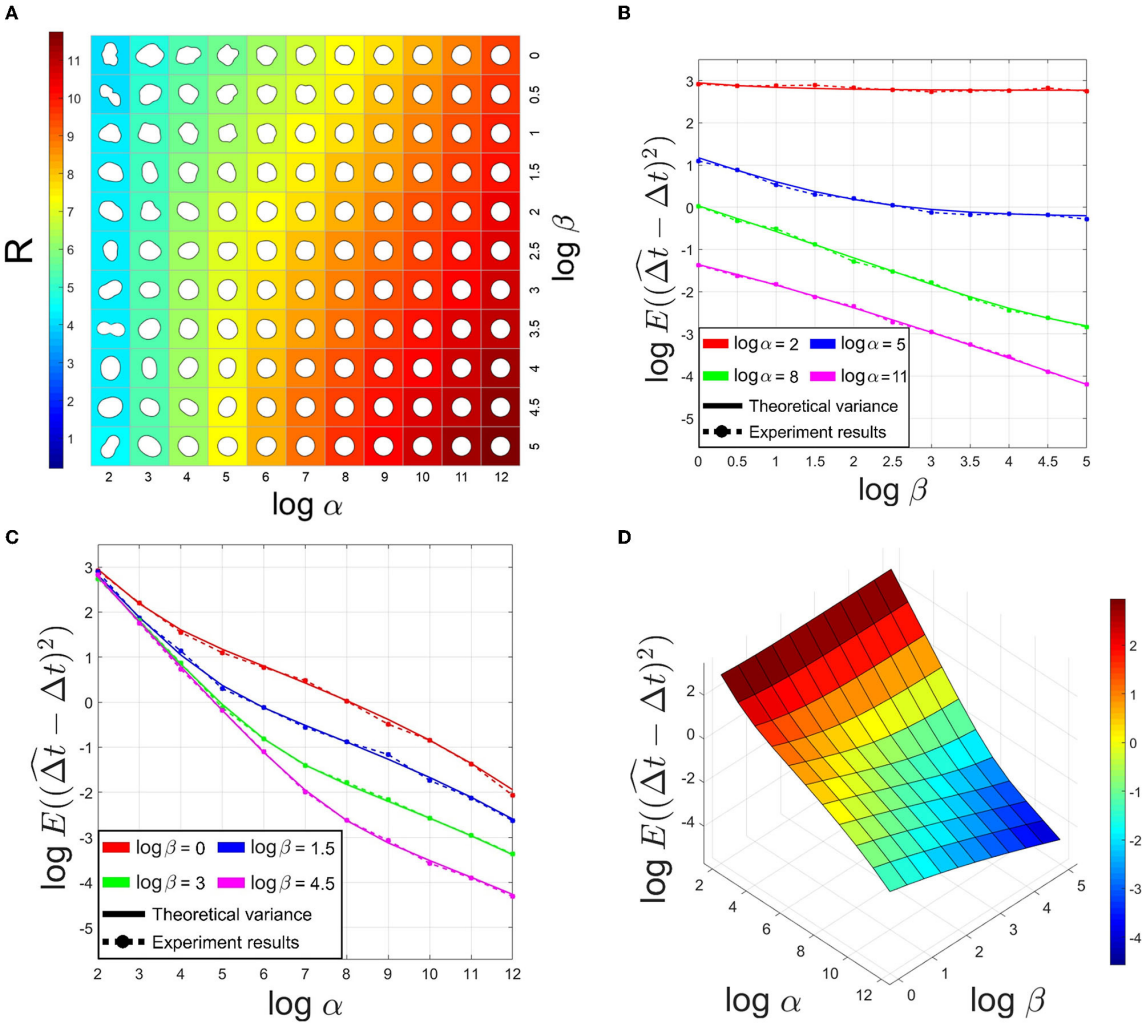
**(A)** Simulated projections of the spherical deformation model with *p* = 4 and changing α and β. The background color indicated *R* value. **(B,C)** Comparison of theoretical variance and experiment results of estimated translation in X-axis on synthetic datasets. **(D)** The theoretical variance of estimated translation with *p* = 4 and changing α and β.

As described above, we generated synthetic structures containing displacements to validate registration accuracy theory. The synthetic dataset includes 121 subsets with different α and β. Since in the deformation model (3), the exponential term *p* non-linearly controls the convergence of λ_*n*_ to 0. To better demonstrate the influence of different α and β, we fix *p* to a proper value in all subsets. In Hobolth (2003), the author used this deformation model to fit neurons in the human brain, calculating *p* at [3.6, 4.4]. Here, we choose *p* = 4 for the following experiments. Each subset constitutes 1,000 pairs of generated shapes with $\bar{r}=150$ pixels and thickness *d* = 40 pixels. We assume that the length of a pixel in the synthetic dataset corresponds to 1 *nm* in the imaging process. Thus, the above sizes in experiments correspond to a regular neuronal structure size and normal section thickness in ssTEM. The translation between images is (4.5 pixels, 4.5 pixels). Figure 2A illustrates the influence of α and β on the synthetic shapes.

### 2.2. Registration Accuracy Analysis

In order to explore the influence of different morphological structures in nerve slices on image registration, we use the spherical deformation model mentioned above to simulate the neural morphological structure. Then, we analyze the registration accuracy between two images containing projections of a local structure.

Consider source image *S* and template image *T* in registration. These two images are different cross-sections of local structure *K* with a shift introduced by the slicing and imaging process, (Δ*t*, Δ*s*). In registration, we first extract *N* corresponding landmarks *l*_*ik*_ = (*x*_*ik*_, *y*_*ik*_), *k* = {*S, T*} in image *S* and image *T*. Landmarks are sampled at an equal angular interval on contours relative to the selected origin. Next, we estimate the translation based on the given cost function. The cost function in registration is as follows


(4) $$\begin{array}{l} \overset{N}{\underset{i=1}{\sum }}{\left(x_{iT}-\left(x_{iS}+u\right)\right)}^{2}+{\left(y_{iT}-\left(y_{iS}+v\right)\right)}^{2}, \end{array}$$

where *u* and *v* are the translation on X-axis and Y-axis, respectively. We align *S* and *T* by estimating the translation $\left(\hat{\Delta t},\hat{\Delta s}\right)$, which minimizes the cost function. Since we consider translation-only registration, landmark *l*_*iT*_ is corresponding to landmark *l*_*iS*_, which are both sampled at the same longitude θ_*i*_. We use ρ_*ik*_ to represent the distance from the origin to the *i*th landmark *l*_*ik*_. ρ_*i*_ is actually the projection of unnormalized radius-vector on the plane, which means $\rho_{ik}=r\left(\theta_{i},\phi_{k}\right)\bar{r}\sin\phi_{k}$. As mentioned above, researchers generally use ultra-thin sections of 40−50*nm* for volume reconstruction. As a result, the size of neuronal structures in adjacent sections are approximately identical, i.e., the mean distance ${\bar{\rho }}_{T}$ from the origin to the neuronal structure contour in *T* roughly equals the mean distance ${\bar{\rho }}_{S}$ in *S*. This relation can be written as *E*(ρ_*iT*_) ≈ *E*(ρ_*iS*_), and can be simplified to sinϕ_*T*_ ≈ sinϕ_*S*_. In our analysis and experiments, we suppose sinϕ_*T*_ = sinϕ_*S*_ = sinϕ.

By computing the partial derivative of the cost function concerning *u* and making the derivative function be zero, we get


$$\begin{array}{l} \hat{\Delta t}=\frac{1}{N}\overset{N}{\underset{i=1}{\sum }}\left(x_{iT}-x_{iS}\right)=\frac{1}{N}\overset{N}{\underset{i=1}{\sum }}\left({\bar{x}}_{T}+\rho_{iT}\cos\theta_{i}-{\bar{x}}_{S}-\rho_{iS}\cos\theta_{i}\right)\\ =\Delta t+\frac{\bar{r}\sin\phi }{N}\overset{N}{\underset{i=1}{\sum }}\cos\theta_{i}\left(r_{iT}-r_{iS}\right)\;, \end{array}$$

where ${\bar{x}}_{k}$ is the X-axis coordinate of the origin in image *k*, and ${\bar{x}}_{T}={\bar{x}}_{S}+\Delta t$. The second-order moment of $\left(\hat{\Delta t}-\Delta t\right)$ is as follows


(5) $$\begin{array}{l} E\left({\left(\hat{\Delta t}-\Delta t\right)}^{2}\right)=\frac{{\bar{r}}^{2}{\left(\sin\phi \right)}^{2}}{N^{2}}E\left(\sum_{i=1}^{N}\sum_{j=1}^{N}\cos\theta_{i}\cos\theta_{j}(r_{iT}-r_{iS})(r_{jT}-r_{jS})\right)\\ \;=\frac{{\bar{r}}^{2}{\left(\sin\phi \right)}^{2}}{N^{2}}\sum_{i=1,j=1}^{N}2\cos\theta_{i}\cos\theta_{j}({\text{Cov}}_{iTjT}-{\text{Cov}}_{iSjT})\\ \;=\frac{{\bar{r}}^{2}{\left(\sin\phi \right)}^{2}}{N^{2}}\sum_{n=2}^{\infty }\lambda_{n}{\left(k_{n}^{0}\right)}^{2}n\\ \;\sum_{i=1,j=1}^{N}2\cos\theta_{i}\cos\theta_{j}\left(P_{n}(\cos\psi_{iTjT})-P_{n}(\cos\psi_{iSjT})\right) \end{array}$$

where Cov_*ik*_1_*jk*_2__ = Cov(*r*(θ_*i*_, ϕ_*k*_1__), *r*(θ_*j*_, ϕ_*k*_2__)). The above analysis is the same as $\hat{\Delta s}$ in the Y-axis. Equation (5) shows that the critical factor affecting the estimation accuracy is the covariance λ_*n*_ when the sampling interval and section latitude are fixed.

Consider the relationship between λ_*n*_ and neuronal structure shape. Now, we analyze why a circle-like projection is more likely to correspond to a more accurate registration result. According to the method of estimating λ_*n*_ based on the Fourier coefficients of a structure projection at polar latitude π/2 in Hobolth (2003), we apply a Fourier transform to the radius-vector function of a projection at polar latitude ϕ_0_, the radius-vector function in terms of the Fourier basis is written as


$$\begin{array}{l} r\left(\theta ,\phi_{0}\right)=\frac{b_{0}}{\sqrt{2\pi }}+\overset{\infty }{\underset{n=1}{\sum }}\left(\frac{b_{n}^{c}}{\sqrt{\pi }}\cos n\theta +\frac{b_{n}^{s}}{\sqrt{\pi }}\sin n\theta \right)\;, \end{array}$$

and the Fourier coefficients $b_{n}^{s}$ of sin*nθ* are given by


$$\begin{array}{l} b_{n}^{s}=\frac{\sin\phi_{0}}{\sqrt{\pi }}\int_{0}^{2\pi }r\left(\theta ,\phi_{0}\right)\sin n\theta \text{ d}\theta \;. \end{array}$$

Under the spherical deformation model, the Fourier coefficient can be modeled as a random variable:


$$\begin{array}{l} b_{n}^{s}=\frac{\sin\phi_{0}}{\sqrt{\pi }}\int_{0}^{2\pi }r\left(\theta ,\phi_{0}\right)\sin n\theta \text{ d}\theta \\ =\frac{\sin\phi_{0}}{\sqrt{\pi }}\int_{0}^{2\pi }\overset{\infty }{\underset{l=2}{\sum }}\overset{l}{\underset{m=-l}{\sum }}a_{l}^{m}\varphi_{l}^{m}\left(\theta ,\phi_{0}\right)\sin n\theta \text{ d}\theta \\ =\sqrt{\pi }\sin\phi_{0}\overset{\infty }{\underset{l=n}{\sum }}k_{l}^{n}P_{l}^{n}\left(\cos\phi_{0}\right)a_{l}^{n}\sim N\left(0,\kappa_{n}\right),\\ \kappa_{n}=\pi \sin^{2}\phi_{0}\overset{\infty }{\underset{l=n}{\sum }}{\left(k_{l}^{n}P_{l}^{n}\left(\cos\phi_{0}\right)\right)}^{2}\lambda_{l},\;n\geq 2. \end{array}$$

The above analysis is also applicable to the Fourier coefficient of cos*nθ*. Because of the characteristics of variance (3), λ_*l*_ decreases rapidly. In Hobolth (2003), the author found that κ_*n*_ is almost only related to the first item λ_*n*_. We simplify κ_*n*_ as follows:


(6) $$\begin{array}{l} \;\kappa_{n}\approx C_{l}^{n}\lambda_{n},\\ C_{l}^{n}=\pi \sin^{2}\phi_{0}{\left(k_{l}^{n}P_{l}^{n}(0)\right)}^{2},n\geq 2. \end{array}$$

Since $b_{n}^{s}$ and $b_{n}^{c}$ obey the same gaussian distribution, we have


(7) $$\begin{array}{l} b_{n}={\left(b_{n}^{c}\right)}^{2}+{\left(b_{n}^{s}\right)}^{2}\sim \kappa_{n}\chi^{2}\left(2\right),\;n\geq 2. \end{array}$$

We can get a good enough interval of λ_*n*_ with the interval estimation method by selecting a reasonable confidence level α. The estimated bound of λ_*n*_ is *b*_*n*_ multiplied by a constant *C*_*n*, α_ determined by α. Therefore, a larger *b*_*n*_ often corresponds to a larger λ_*n*_. The accuracy of the bound of λ_*n*_ is determined by α. By Equations (6) and (7), a larger *b*_*n*_ is more likely to correspond to a larger λ_*n*_. We construct an index *R* to reflect the theoretical registration accuracy,


(8) $$\begin{array}{l} R=-\log\left(\overset{N}{\underset{n=1}{\sum }}b_{n}\right)\;. \end{array}$$

On the one hand, based on the above analysis, we know that the larger *R* is, the larger λ_*n*_ is likely to be, and with Equation (5), the registration accuracy is expected to be worse. On the other hand, since *R* is an index related to the Fourier coefficients, *R* reflects whether a shape is close to a circle or not. We validate the mean of *R* on the synthetic dataset, as Figure 2A shows. The results show *R* increases with the shape roundness. According to Figure 2A, we select *R*_*thres*_ = 6 as a threshold to judge whether a local structure is a "sphere-like" object in the following process. The other three results in Figure 2 demonstrate that registration accuracy is positively correlated with shape roundness, just as above analysis.

### 2.3. Serial Neural Image Registration

Based on the above neuronal morphological modeling analysis, we further propose a novel framework for serial neural image registration, which utilizes the characteristics of the anatomical structure of nerve tissue to obtain a more reasonable corresponding relationship. The proposed image registration method for serial EM sections is presented in three parts: neuronal morphological model-driven region selection, correspondence extraction and global optimization, and image deformation.

#### 2.3.1. Neuronal Morphological Model-Driven Region Selection

On the basis of Section 2.2, more regular circular regions in the section image of neural tissue should be selected to extract correspondences. Hence, in this part, we try to obtain a mask of neural images that focus on the stable structures and pay less attention to the variant structures. First, we utilize the deep membrane segmentation network "FusionNet (Quan et al., 2016)" and the traditional region segmentation algorithm watershed (Bieniek and Moga, 2000) to segment all neural structures. We then use the neural morphological physical selection model to pick the stable structures. Through the segmentation method and neural morphological physical selection model, a mask is learned to focus on the stable region and pay less attention to the changing regions (unstable structure).

FusionNet is widely used in cell membrane segmentation. Similar to the traditional U-Net framework, Fusionet includes a contraction path to extract features and a symmetrical expansion path to better locate. Furthermore, it embeds a residual module between lower sampling and upper sampling in each layer and retains the layer hopping connection of U-Net. As a result, it has more robust advantages than U-Net in neuron boundary extraction (Quan et al., 2016). Therefore, in this paper, we adopt the Fusionnet structure. The number of output feature channels of the first residual block of the network is 32, and the number of feature channels increases two times every subsequent sampling. In addition, there are four lower sampling layers. Similarly, there are four upper sampling layers. The number of feature channels is reduced by two times every upper sampling. The last layer of the network is the sigmoid function to ensure that the range of the final output probability diagram is between 0 and 1. Our loss function adopts the single-pixel MSE function, which can ensure that the width of the output cell membrane will not be thicker or thinner than the ground truth. The training dataset is from the ISBI two-dimensional electron microscopy segmentation challenge (Arganda-Carreras et al., 2015), which included a 2 × 2 × 1.5 *um*^3^ volume imaged from 30 sections and publicly available manual segmentations. During instance testing, the corresponding binary image of the cell membrane can be obtained. The watershed algorithm (Bieniek and Moga, 2000) is used for different segment cells in the process, which takes the similarity between adjacent pixels as a reference so that the pixels with similar spatial positions and similar gray values are connected to form a closed contour. Finally, the desired more rounded regions are selected by the morphological threshold *R*_*thres*_. Algorithm 1 describes an implementation of the complete model-driven region selection process.

**Algorithm 1:** Region selection.

**Input:** EM Image *I*
1: Membrane Segmentation: *M*_*res*_ ← FusionNet(*I*)
2: Structure Segmentation: *S*_*res*_ ← Watershed(*M*_*res*_)
3: Number of Structures: *N*_*res*_ ← CountStructures(*S*_*res*_), *i*←0
4: **repeat**
5: *i*←*i* + 1
6: Get *i*th Structure *S*_*i*_ in *S*_*res*_
7: **if** *R*(*S*_*i*_) < *R*_*thres*_, *R* is based on Equation (8) **then**
8: Delete *S*_*i*_ in *S*_*res*_
9: **end if**
10: **until** *i*> = *N*_*res*_
11: *M* ← *S*_*res*_
**Output:** Region Mask *M*

#### 2.3.2. Correspondence Extraction and Global Optimization

After the specific structural regions were selected, we adopt SIFT-flow (Liu et al., 2011) to extract the reliable correspondences in these areas, assuming that the structure of the biological tissue changes independently, which means that the angle of the structure to the section plane is random. As an optical flow method, SIFT-flow searches the correspondence for every pixel. Pixelwise SIFT features between the adjacent section *i* and *i* + 1 are matched as a discrete optimization problem,


(9) $$\begin{array}{l} \begin{array}{c} E\left(w_{i}\right)=\underset{p_{i}}{\sum }min\left(\|s_{i}\left(p_{i}\right)-s_{i+1}\left(p_{i}+w_{i}\right)|{|}_{1},t\right)\\ +\underset{p_{i}}{\sum }\eta \left(\left|u\left(p_{i}\right)|+|v\left(p_{i}\right)\right|\right)\\ +\underset{\left(p_{i},q_{i}\right)\in \varepsilon }{\sum }min\left(\alpha |u\left(p_{i}\right)-u\left(q_{i}\right)|,d\right)\\ +min\left(\alpha |v\left(p_{i}\right)-v\left(q_{i}\right)|,d\right) \end{array} \end{array}$$

where *w*_*i*_(*p*_*i*_) = (*u*(*p*_*i*_), *v*(*p*_*i*_)), and *s*_*i*_(*p*_*i*_) are the displacement vector and the SIFT descriptor at location *p*_*i*_ in section *i*, respectively, ε contains all the spatial neighborhoods. The third term in function 9 is used as a smoothness constraint, which constrains the adjacent pixels to have similar displacement. With the assumption that the neighboring structures of the biological tissue change independently, the extracted correspondences between the adjacent sections are more reliable compared with SIFT and block-matching methods.

Although all of the pixels in the selected regions of the adjacent sections can be used as correspondences, the vertexes of a grid placed in the section are selected due to computational burden. The positions of the extracted correspondences need to be adjusted so that the points of each correspondence at the adjacent sections have the same positions in the *xy* plane. To avoid error accumulation, the correspondences through all of the sections are adjusted simultaneously. In addition to the position consistency of the correspondences, the displacements of these correspondences are constrained to be smooth and small, which restricts the nonlinear deformation of the original images. An energy function with an equality constraint for the sections (*i* = 1, ⋯ , *n*) is utilized to calculate the displacement of the correspondences.


(10) $$\begin{array}{l} \begin{array}{c} E\left(w_{i},\cdots \;,w_{i},\cdots \;,w_{n}\right)=\underset{i}{\sum }\underset{p_{i}}{\sum }\left(u{\left(p_{i}\right)}^{2}+v{\left(p_{i}\right)}^{2}\right)+\\ \underset{i}{\sum }\underset{\forall \left(p_{i},q_{i}\right)}{\sum }\frac{\lambda }{dist\left(p_{i},q_{i}\right)}\left({\left(u\left(p_{i}\right)-u\left(q_{i}\right)\right)}^{2}+{\left(v\left(p_{i}\right)-v\left(q_{i}\right)\right)}^{2}\right)\\ s.t.\;p_{i}+w_{i}=p_{i+1}+w_{i+1}\;for\;every\;correspondence\;\left(p_{i},p_{i+1}\right) \end{array} \end{array}$$

where *w*_*i*_(*p*_*i*_) = (*u*(*p*_*i*_), *v*(*p*_*i*_)) is the displacement vector at location *p*_*i*_ in section *i*, and *dist*(*p*_*i*_, *q*_*i*_) is the Euclidean distance between *p*_*i*_ and *q*_*i*_. The equality constraint keeps the position consistency of the correspondences between the adjacent sections and avoids the value of the displacement vector *w*_*i*_ in the energy function to be zero.

#### 2.3.3. Image Deformation

With the displacement vector of the extracted correspondences, we have the positions of the points in the original section image and their positions in the aligned image for each section. Any image deformation method based on the control points could be used to transform the original section into the aligned section. Here, the moving-least squares (MLS) method (Schaefer et al., 2006) is used to warp each section image. The deformation result produced by the MLS method is globally smooth, and as a result of using rigid transformation, rigidity and scale are maintained locally so that the biological tissue can retain its local shape as rigidly as possible.

## 3. Experiments and Results

In this section, to illustrate the proprieties of our proposed framework, we tested the effect of sub-modules: neuronal morphological model-driven region selection module and correspondence extraction and global optimization module. Furthermore, we performed volume reconstruction experiments on the serial section neural image datasets to demonstrate the capability of the whole proposed framework.

### 3.1. Neuronal Morphological Model-Driven Region Selection

**Figure 3:**
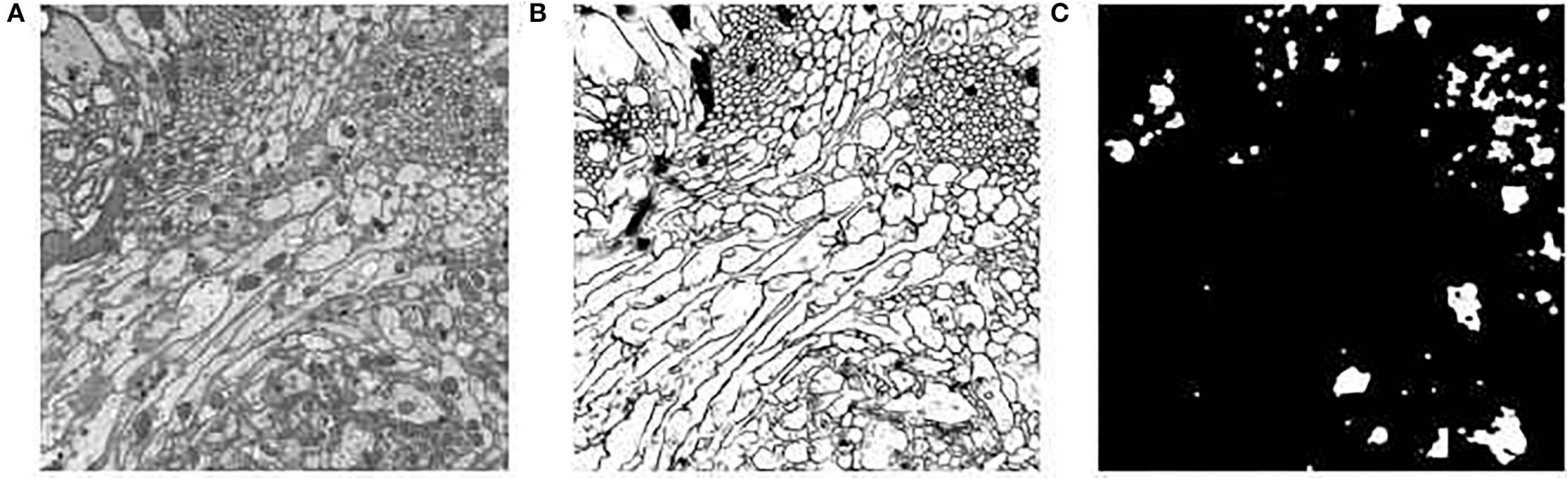
The results of extracting membrane boundaries and the selection of the regular circular region: **(A)** original image; **(B)** the results of membrane segmentation; **(C)** the results of region selection.

We assessed the efficiency of the neuronal morphological model-driven region selection. We implemented the segmentation network using Keras and used a GPU workstation equipped with a NVIDIA 2080 Ti GPU. The neural cell membrane segmentation network is trained and verified on the ISBI2015 dataset (Arganda-Carreras et al., 2015), which is from Drosophila brain imaging by FIB-SEM and has the ground truth of membrane segmentation results. We got 95% membrane segmentation accuracy in the test dataset. Then, we apply the network parameters to DFS dataset, which has the same image style and could directly utilize our pre-trained model. The images from DFS data are cut to a smaller size of 512 × 512 to suit the network input. The membrane segmentation result is shown in Figure 3B. After that, the watershed algorithm is utilized to separate the regions of neural structure. Then, through region selection guided by the neuronal morphological model, we could get our desired stable region mask as shown in Figure 3C. We could see that the selected regions are relatively regular and concentrated in the nerve fiber bundle areas.

**Figure 4:**
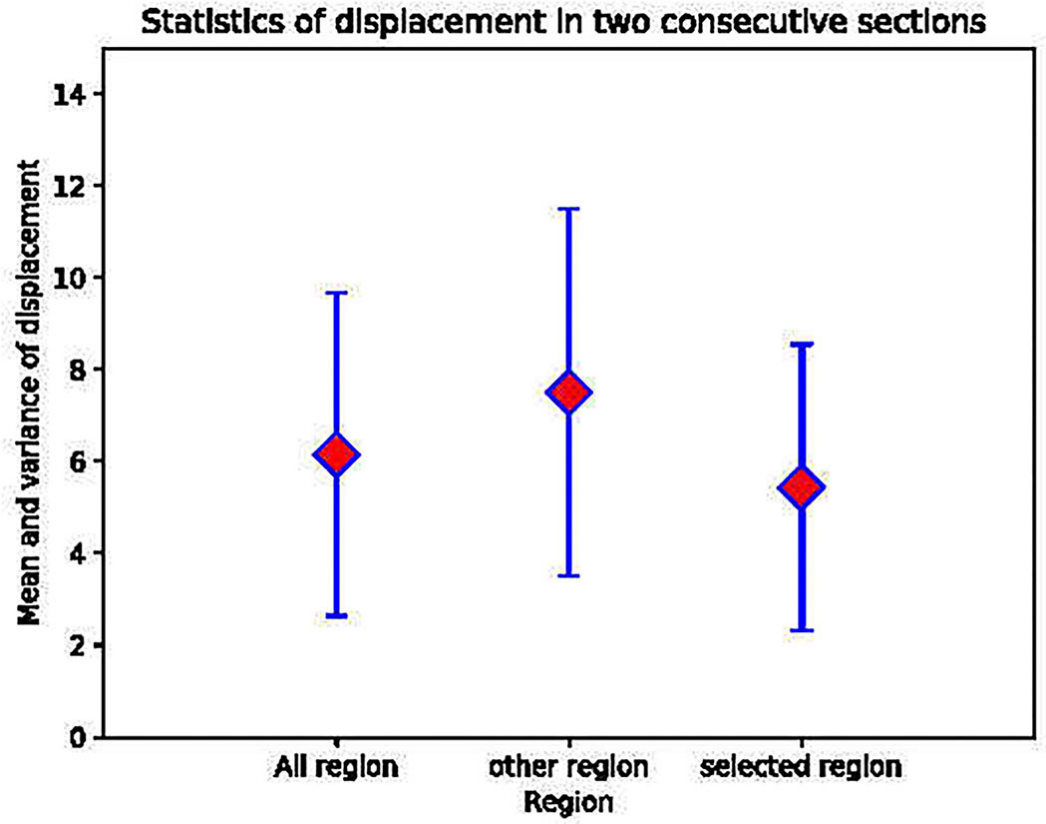
The mean and the variance in the displacement between the regular circular areas, other areas and the whole image in two consecutive layers.

To further verify that the selected areas are indeed the more stable areas, we performed experiments using DFS dataset to measure the registration accuracy. DFS dataset are imaged *in situ* and the corresponding point is the direct correspondence of the pixel position. We calculated the position deviation of the corresponding points of the mask areas, the areas outside the mask, and the overall image. The calculation results showed that the mean and variance in displacement calculated in our mask regions are smaller than other regions, as shown in Figure 4. This proves that the regular circular areas change slowly and verifies our hypothesis that the stable regions extracted are more effective for improving registration accuracy.

### 3.2. Correspondence Extraction and Global Optimization

We quantitatively evaluated the accuracy of the correspondence detection methods on adjacent sections acquired by DFS dataset. As the sections are imaged *in situ*, we can measure the absolute distances of the extracted correspondences as the ground truth. Among the 30 pairs of adjacent images in DFS dataset, we randomly select five starting coordinates for cutting image pairs. A total of 150 image pairs with 512 × 512 pixels are acquired for evaluating the accuracy of the correspondences. Our method was compared with two classical methods on correspondence detection, block matching and SIFT. Block matching method uses intensity information to search the correspondences. In some previous reconstruction works, block matching method was often used for slice image alignment (Saalfeld et al., 2012). SIFT is a stable local feature descriptor, maintaining invariant to rotation, scale scaling, brightness change (Lowe, 2004).

**Figure 5:**
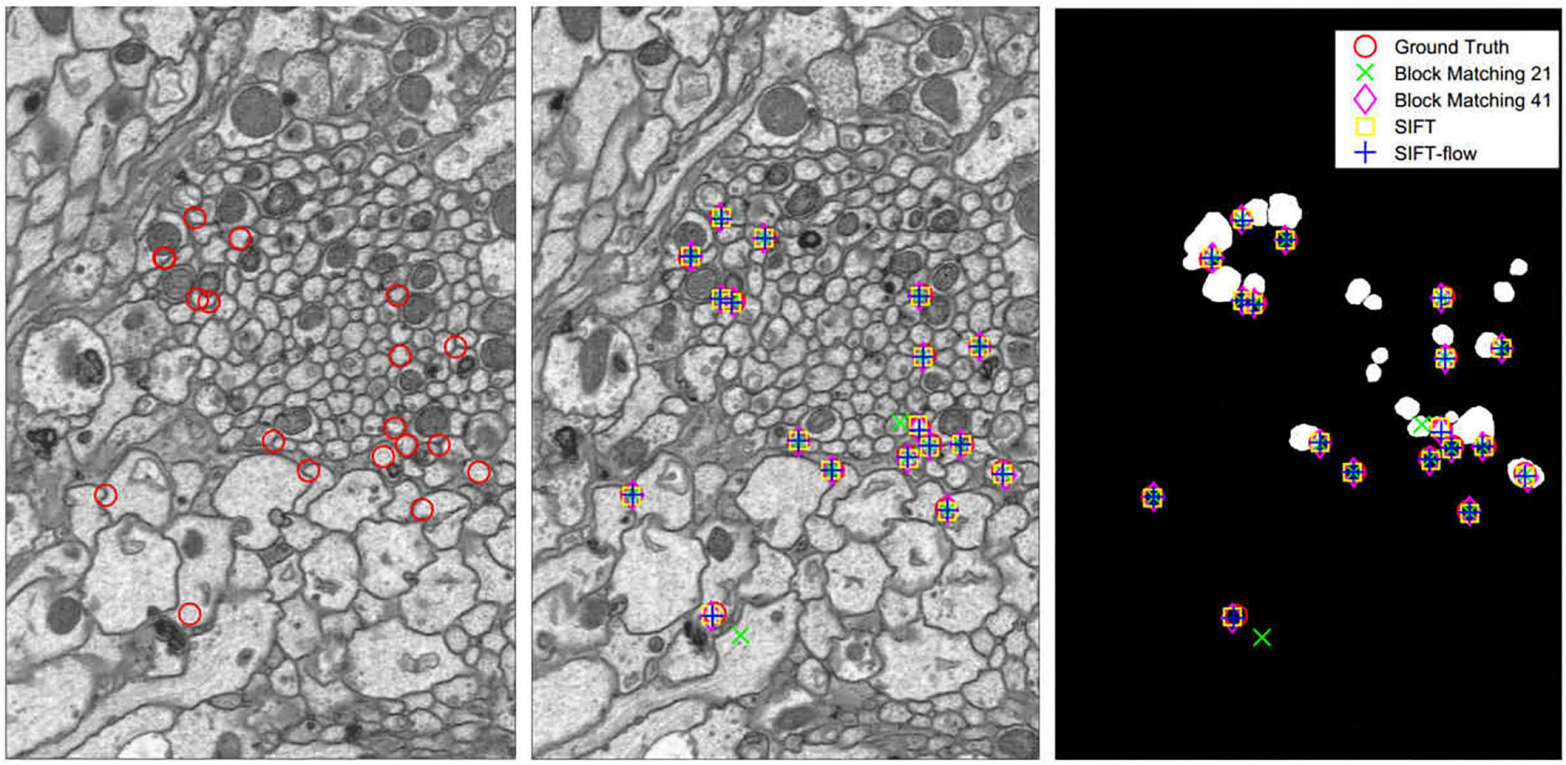
Left: the points in the reference section. Middle: the correspondences extracted in the adjacent section. Right: the correspondences extracted in the selected region map.

**Figure 6:**
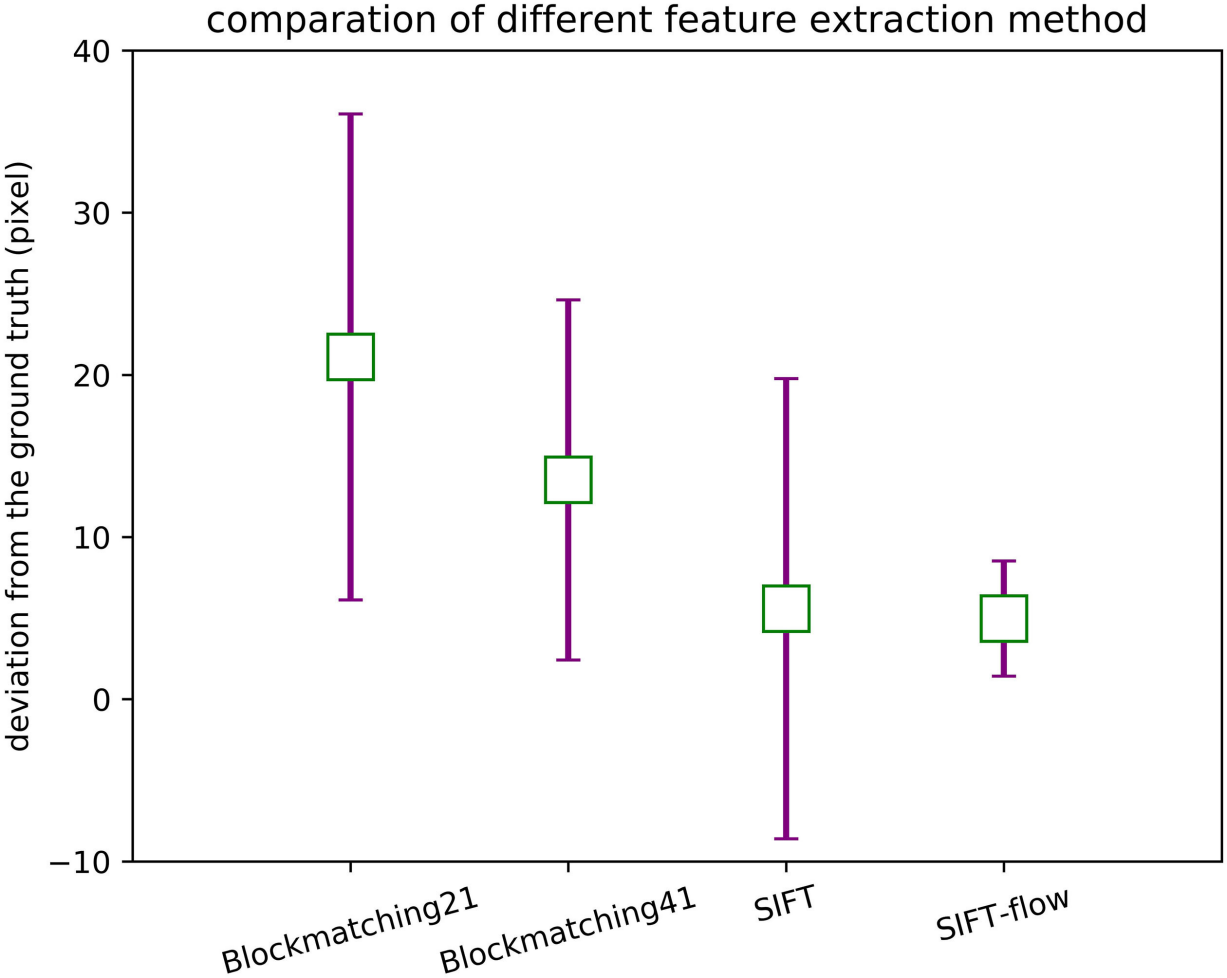
The mean and the variance in the distances between the extracted correspondences and the ground truth.

We tested block-matching methods with block size 21 × 21 and 41 × 41, SIFT and SIFT-flow. SIFT detects correspondences, and the points in the reference section are selected to detect their correspondences in the other section by block matching and SIFT-flow. Figure 5 displays the comparison of the corresponding relationship extraction of image pairs. The mean and the variance in the distances between the extracted correspondences and the ground truth of 150 image pairs are shown in Figure 6. As expected, most correspondences lie in the selected regions, and the SIFT-flow method achieves the best result because the extracted correspondences are calculated considering the neighborhood information.

### 3.3. Evaluation of Serial Section Electron Microscope Image Dataset

The registration of the serial section recovers three-dimensional continuity and reconstructs the 3*D* structure of biological tissue. To test the effect of restoring the z-axis continuity of neural structure, we employed our proposed method on the serial neural section images datasets: DST and DFS. Both the subjective and objective evaluation criteria are considered as methods to evaluate the results of serial section image registration methods. We also employed our proposed method on the DST-CREMI dataset for reasonably comparing with the learning-based method ssEMNet (Yoo et al., 2017).

**Figure 7:**
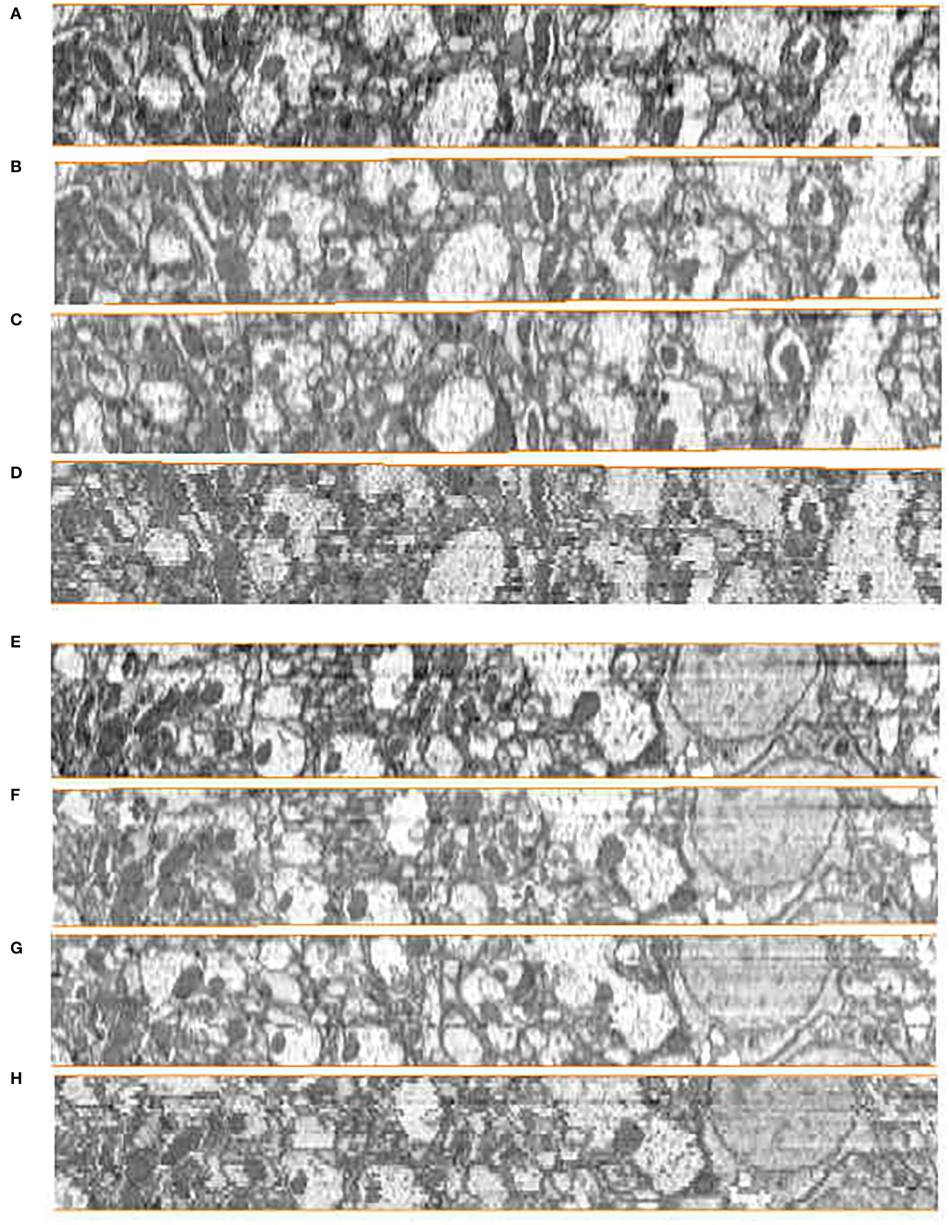
Registration results in the *yz* plane on the Drosophila FIB-SEM (DFS) dataset. **(A,E)** Ground truth. **(B,F)** Our results. **(C,G)** Wang CW results. **(D,H)** Elastic results.

Subjective evaluation is usually conducted by experienced technicians according to certain rules to score. In serial neural image registration, this scoring evaluation depends on the restoration of the continuity of the nerve structure in the Z direction after registration. To visualize the continuity performance of serial registration methods, we stack the serial 2D images into volume and get the Z-axis side view of the alignment results. The side views of the reconstructed anatomical object of the DFS dataset by the ground truth and the compared methods are presented in Figure 7. We compared our proposed method with the state-of-the-art serial section registration methods: Wang CW's (Wang et al., 2015) and Elastic methods (Saalfeld et al., 2012). It is shown that the proposed method and Wang CW's method achieve solid reconstructed objects with less discontinuity. While block matching-based elastic method has more shaky alignment results than others. These are mainly caused by the differences in the ability to extract the corresponding relationships. The DFS datasets with 100 nm slice thickness has large changes in the content of adjacent slice images. Due to the limited feature expression ability of the block matching method, the elastic method easily gets the incorrect correspondences when dealing with intensity changing images, leading performance degradation. On the contrary, Wang CW's and our proposed method utilizing SIFT with stronger presentation ability can better handle this. Compared with Wang CW's and our proposed method, the results of our proposed method can better maintain the morphology of neural structure. The correspondence constraint item of our proposed method based on the flow field model can punish those abnormal matching relationships, so as to reduce the matching error and improve the accuracy of feature matching. What is more, the correspondences extraction in line with the selection of neural morphological structure can cope with the changes in image contents from biological tissue. Hence, better registration results are acquired by our proposed method.

We also counted the evaluation of registration results by several experienced technicians. We produced a questionnaire that contains the registration results of different methods. The data of subjective evaluation are prepared as follows: the aligned sections are placed sequentially on the z-axis, and the anatomical object of the biological tissue is reconstructed by interpolating along the z-axis to match the pixel size in the *xy* plane. The reconstructed anatomical object should have a smooth neuron membrane and mitochondria with a solid border, which is evaluated subjectively on the randomly selected side view plane by five experienced technicians for labeling electron microscopy data. Each person has 20 images of each dataset, and the total number of the to be evaluated images of each dataset is 100. Each person can only choose the best method from the results of various registration methods for each dataset, and the method score of the dataset is increased by 1. The statistical scoring results by experienced technicians are shown in Table 1. It is obvious that our proposed method has the highest score. To some extent, it can also show that our registration effect has better visual continuity.

**Table 1:** The scores of the subjective evaluation of Drosophila ssTEM (DST) and Drosophila FIB-SEM (DFS) dataset.

**Dataset**	**Method**		
	**Elastic results**	**Wang results**	**Our results**
DST dataset	2	33	**65**
DFS dataset	0	37	**63**

*The highest values are marked in bold*.

**Figure 8:**
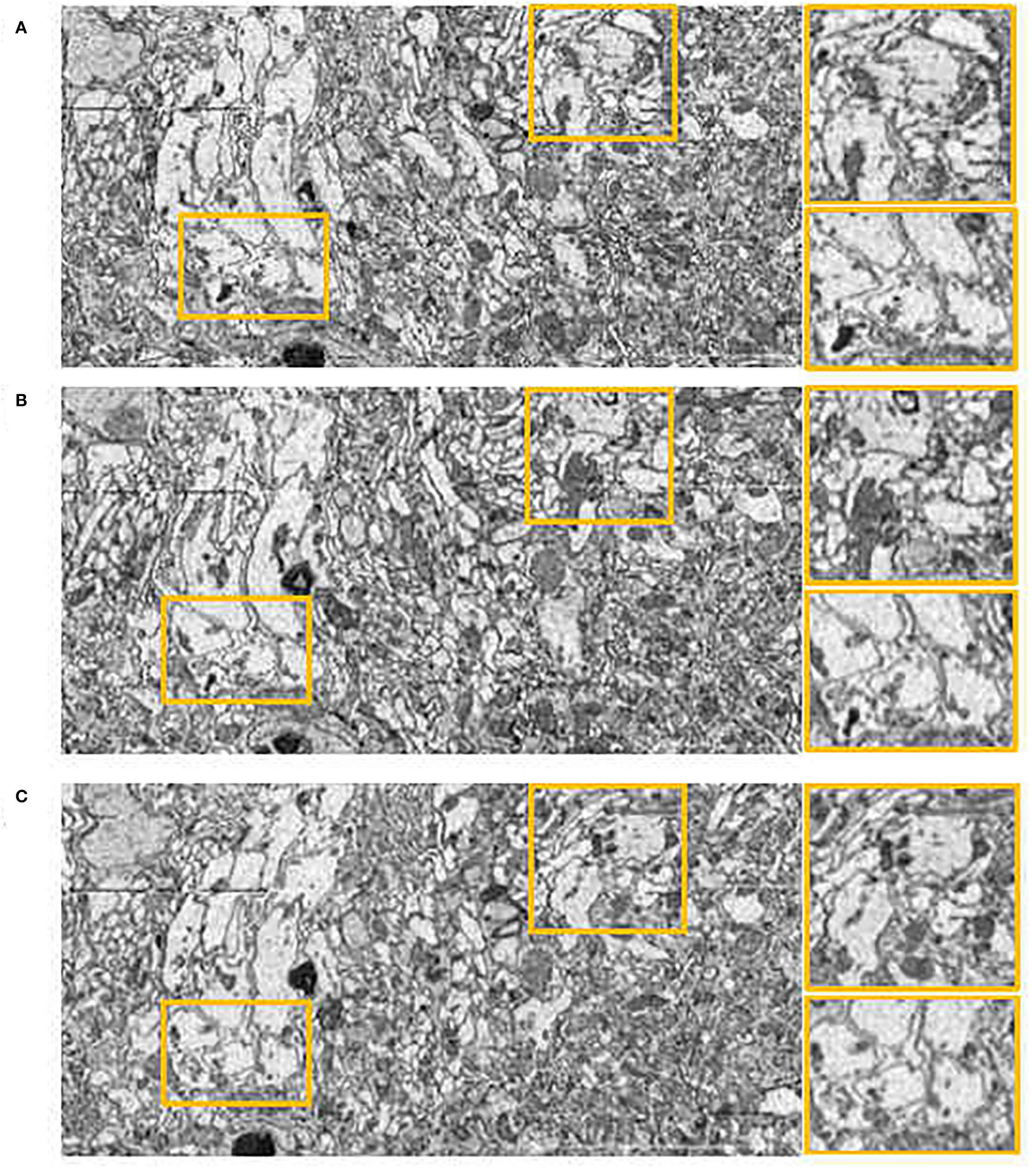
Registration results in *yz* plane on the Drosophila ssTEM (DST) dataset. **(A)** Wang CW results. **(B)** Elastic results. **(C)** Our results.

In order to further assess the effectiveness of our method on other datasets, we evaluated our method, Wang CW's method (Wang et al., 2015) and Elastic method (Saalfeld et al., 2012) on DST dataset. Compared with the experimental settings on the DFS dataset, the Elastic method had different parameter settings due to the different slice thicknesses (100 *nm* in the DFS dataset, 40−50 *nm* in the DST dataset). The hyperparameters of elastic method are block matching search radius and block radius. After testing, we set 100 pixels block matching search radius, 20 pixels block radius in DST dataset, while 200 pixels,40 pixels in DFS dataset. The side views of the reconstructed anatomical object of the DST dataset by these methods are presented in Figure 8. The proposed method still achieves the best visual effect considering continuity. We also notice that compared with the results in the DFS dataset, the result of the Elastic method in Figure 8 looks significantly less shaky. Wang CW's method and our proposed method do not suffer from inconsistent alignment results on the used dataset. We find that the difference in alignment resulting in a different dataset may be related to the slice thickness. The difference between adjacent images increases as the slices thickness increases. As mentioned earlier, based on the feature selection and extraction of neural morphological structure, combined with the constraints of global optimization, our proposed method has a more stable alignment performance when dealing with such intensity changing images. This also proves that our method has better adaptability to different datasets.

**Figure 9:**
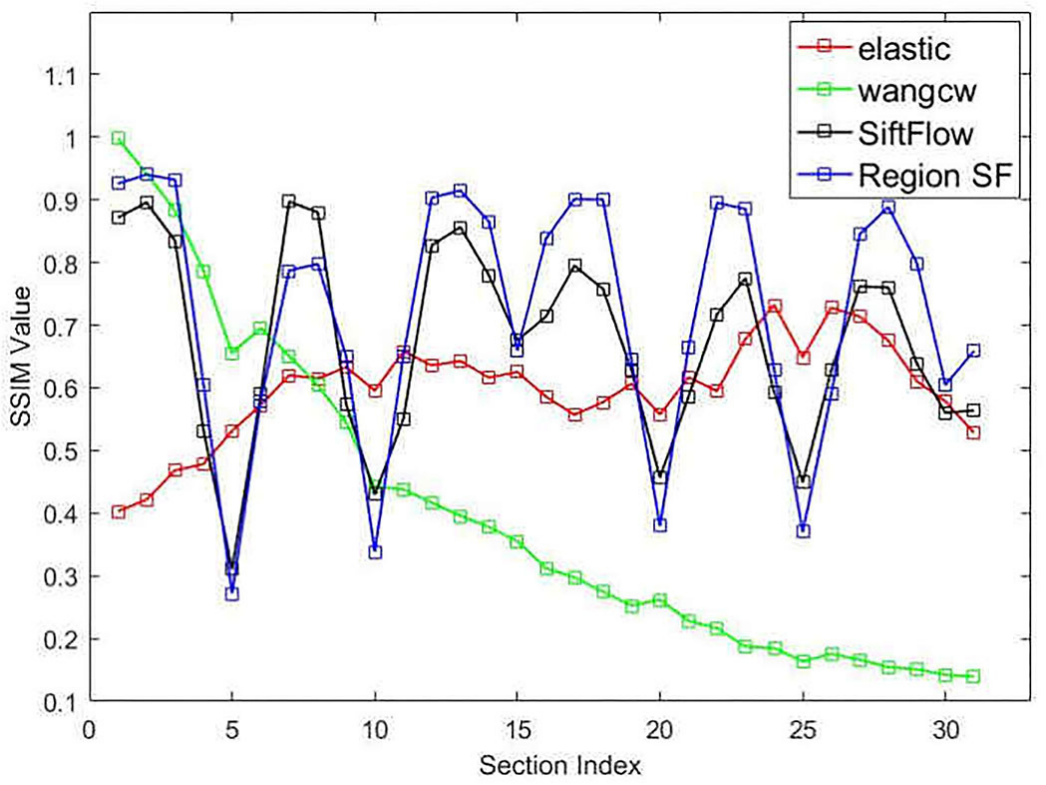
Structural similarity index (SSIM) between the aligned sections and the ground truth.

The structural similarity index (SSIM) (Wang et al., 2004) is usually adopted to quantitatively measure the registration accuracy as an objective evaluation criterion. We also computed this objective evaluation criterion for the DFS dataset to quantitatively evaluate the serial registration methods. Figure 9 presents the quantitative evaluation results of individual approaches, the vertical axis represents the structural similarity between each transformed source image and ground truth after serial image registration. We annotated our proposed neuronal morphological model-driven serial image registration method as "Region SF" for better plotting figures. As the section index increases, the score of the Wang CW's method declines obviously because of error accumulation and propagation, which means that artificial deformation of the images far away from the reference image is aggravated. We notice that elastic method shows higher consistency than other methods, for which we thank the effective constraint of the elastic mesh model in serial registration. However, elastic mesh model constraint also restricts the ability to process abnormal slices. When large deformation occurs (slice damage or wrong correspondence), the elastic method will propagate this abnormal condition under the transmission of spring, leading to the poor performance of the elastic method in Figure 9. Our proposed neuronal morphological model-driven serial image registration method has better SSIM value and retains the morphology of the original section images better than the comparing methods, except for the sections that suffered from the artificial generated distortion. Although some distortions exist in the sections, most of the section images still reflect the real 3D structure of the sectioned biological tissue, which proved that our proposed method could retain as much as the morphology of the acquired serial EM section images as possible.

In addition, we used the open-source dataset DST-CREMI for a reasonable comparison of our proposed method and the learning-based serial section image registration methods ssEMNet (Yoo et al., 2017), which receives extensive attention from the serial image registration community. We reproduced ssEMNet according to the parameters mentioned in the original paper and tested it on a workstation equipped with Tesla V100 Graphics Processing Units (GPUs). SSIM index is utilized to quantitatively measure the registration accuracy between the aligned results and the ground truth image and between the aligned results and the adjacent image. The quantitative results in the Table 2 shows that our proposed method achieve the comparable performance compared with ssEMNet and better than elastic and Wang CW's methods on DST-CREMI dataset. The generalization of our method is proved again, which is suitable for section image datasets from different imaging modes and different samples. We could find that our proposed method has a superior SSIM value (with adjacent). Although the feature information output from the deep network has stronger representation ability than the traditional feature descriptor, our method performs better in maintaining the continuity of biological structure by using biological prior information screening the more robust corresponding points.

**Table 2:** Quantitative results comparison of structural similarity index (SSIM) on Drosophila ssTEM (DST)-CREM datasets.

**Method**	**Metric**	
	**SSIM (with GT)**	**SSIM (with adjacent)**
Elastic	0.2763	0.4007
Wang CW's	0.2809	0.5402
ssEMNet	**0.2982**	0.6638
Ours	0.2847	**0.6683**

*The highest values are marked in bold*.

## 4. Conclusion

In this paper, we consider whether there are such stable "markers" in the neural images to alleviate registration difficulty. We employ the spherical deformation model to simulate the local neuron structure and mathematically analyze the relationship between registration accuracy and neuronal structure shape in two adjacent sections by the second-order moment of estimated translation. Through modeling and analysis, we can prove that the registration accuracy is positively correlated with neuronal structure roundness, which is to say, the more regular the structure, the more stable it is, and it is more conducive to registration.

Based on the analysis results of neural morphological modeling, we designed a new serial image registration framework with a structure selection module, utilizing the characteristics of the anatomical structure of nerve tissue to obtain a more reasonable corresponding relationship. The proposed framework consists of a neuronal morphological model-driven region selection module, correspondence extraction and global optimization module, and image deformation module. Our proposed method leverages the deep membrane segmentation network and neural morphological physical selection model to select the changeless rounded regions in neural structure. The output region selected masks combined with feature extraction and global optimization of correspondence position to obtain the deformation field of multiple images.

Compared with the state-of-the-art approaches, the proposed method achieves better genuine deformation results and better quantitative results on serial EM section image datasets. However, we still have some limitations in that utilizing a deep segmentation network selected region and SIFT-flow method extracted correspondences to obtain the deformation field, not a totally end-to-end method. The lack of joint optimization in separate steps may lead to some reduction in registration accuracy. The proposed method is not robust enough when the images have large distortions. In the future, we will explore how to use an end-to-end method to utilize the structural information of ssEM images for better registration.

## Data Availability Statement

The original contributions presented in the study are included in the article/supplementary material, further inquiries can be directed to the corresponding author.

## Author Contributions

FZ and BC designed the experiments and wrote the article. XC and HH conceived the ideas for all the methods and provided suggestions based on the experimental results. All authors contributed to the article and approved the submitted version.

## Funding

This research is supported by the Strategic Priority Research Program of Chinese Academy of Science (No. XDB32030208 to HH), International Partnership Program of Chinese Academy of Science (No. 153D31KYSB20170059 to HH), Program of Beijing Municipal Science & Technology Commission (No. Z201100008420004 to HH), CAS Key Technology Talent Program (No. 292019000126 to XC), Instrument Function Development Innovation Program of Chinese Academy of Sciences (No. E0S92308 to XC).

## Conflict of Interest

The authors declare that the research was conducted in the absence of any commercial or financial relationships that could be construed as a potential conflict of interest.

## Publisher's Note

All claims expressed in this article are solely those of the authors and do not necessarily represent those of their affiliated organizations, or those of the publisher, the editors and the reviewers. Any product that may be evaluated in this article, or claim that may be made by its manufacturer, is not guaranteed or endorsed by the publisher.
